# Multimodal Neural Network for Rapid Serial Visual Presentation Brain Computer Interface

**DOI:** 10.3389/fncom.2016.00130

**Published:** 2016-12-20

**Authors:** Ran Manor, Liran Mishali, Amir B. Geva

**Affiliations:** ^1^Department of Electrical and Computer Engineering, Ben-Gurion University of the NegevBeer-Sheva, Israel; ^2^Department of Electrical and Computer Engineering, Tel Aviv UniversityTel-Aviv, Israel

**Keywords:** EEG, deep learning, BCI, RSVP, single trial, neural network, computer vision

## Abstract

Brain computer interfaces allow users to preform various tasks using only the electrical activity of the brain. BCI applications often present the user a set of stimuli and record the corresponding electrical response. The BCI algorithm will then have to decode the acquired brain response and perform the desired task. In rapid serial visual presentation (RSVP) tasks, the subject is presented with a continuous stream of images containing rare target images among standard images, while the algorithm has to detect brain activity associated with target images. In this work, we suggest a multimodal neural network for RSVP tasks. The network operates on the brain response and on the initiating stimulus simultaneously, providing more information for the BCI application. We present two variants of the multimodal network, a supervised model, for the case when the targets are known in advanced, and a semi-supervised model for when the targets are unknown. We test the neural networks with a RSVP experiment on satellite imagery carried out with two subjects. The multimodal networks achieve a significant performance improvement in classification metrics. We visualize what the networks has learned and discuss the advantages of using neural network models for BCI applications.

## 1. Introduction

Brain-Computer Interfaces (BCI) is a communication method using the brain's electrical activity to control a machine to perform tasks. BCI systems have been extensively researched to assist locked-in patients. However, advances in computing power and algorithms paved way for BCI applications for healthy users as well. In this case, electroencephalography (EEG) is the preferred method for recording brain activity. EEG is a noninvasive method, that records data from multiple electrodes placed on the user's scalp. EEG devices can record data with high temporal resolution from all electrodes simultaneously, generating data matrices that represent the ongoing brain activity. In addition, EEG is mobile and more affordable than other neuroimaging methods, and is thus a common choice for real-life BCI applications. EEG has been used for a wide variety of tasks, such as detecting real or imaginary hands movements (Müller-Gerking et al., 1999) and spelling words (Kaper et al., 2004). One interesting category of BCI applications is the passive BCI (Zander and Kothe, 2011). This category of applications is intended to augment the human-machine interaction using the brain's activity, without the user having to focus on control tasks.

One such passive BCI application is the Rapid Serial Visual Presentation (RSVP) application (Parra et al., 2007; Bigdely-Shamlo et al., 2008; Alpert et al., 2014). In RSVP tasks, a subject is instructed to search and count for target images within a continuous stream of images, displayed at a fast pace, e.g., at 10 Hz. Such applications are useful for classifying large image sets, where on one hand it is too time consuming to do manual classification, and on other the hand computer vision (CV) algorithms do not perform well enough on their own.

BCI applications for healthy subjects, and specifically RSVP tasks, are required to decode brain activity from a single recording of EEG. Single trial EEG data typically contain multiple noise sources, e.g., measurement noise and on-going brain activity which is irrelevant to the task at hand. Therefore, most BCI applications use machine learning (ML) algorithms to learn the task-relevant patterns from the experiment data (Pfurtscheller et al., 2002; Müller et al., 2003; Felzer and Freisieben, 2004; Blankertz et al., 2007; Lotte et al., 2007).

**Figure 1:**
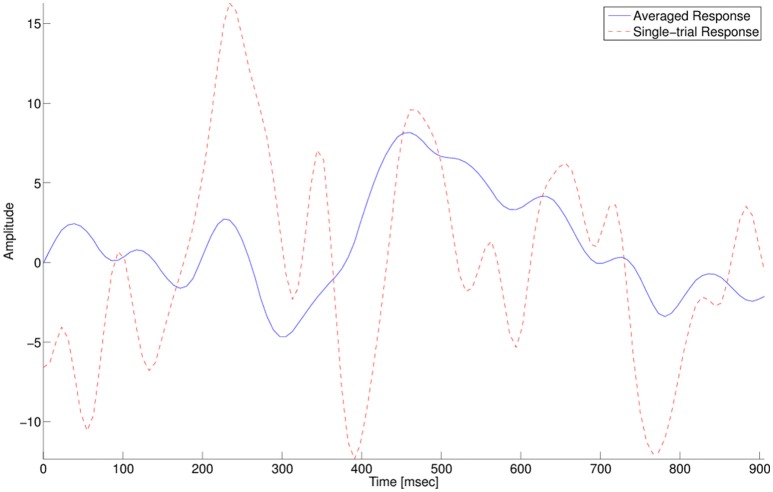
**P300 target-response at electrode Pz**. The solid blue line shows an averaged response of multiple single trials. The dashed red line shows a single trial response.

In RSVP tasks, the goal of the BCI application is to automatically identify single trial spatio-temporal brain responses that are associated with the target image detection, the P300 event-related potential (ERP). The P300 signal is generated when a rare target stimulus is detected among non-target stimuli (Donchin et al., 2002). As its name suggests, the P300 is a positive potential, with latency around 300 ms after the stimulus onset. The ERP is usually visible when several targets responses are averaged, while in a single trial response it is masked by noise and other brain activity, as seen in Figure 1. In addition, the amplitude and latency of the P300 have a large variance between subjects, and within subjects.

RSVP tasks present specific challenges for single trial classification algorithms, mainly due to the fast presentation of stimuli which causes an overlap of consecutive brain responses. Therefore, methods have been constructed specifically for RSVP applications.

One such method, developed by Bigdely et al. (Bigdely-Shamlo et al., 2008) for single-trial classification of RSVP data, uses spatial Independent Component Analysis (ICA) and Principal Component Analysis (PCA) for features extraction. This method extracts spatial, temporal, and spectral features, which are ultimately combined and classified using a Fisher Linear Discriminant (FLD) classifier. Parra et al. (2007) proposed a framework for RSVP experiments. The framework uses bi-linear projections of the EEG data matrix on both temporal and spatial axes, and can be implemented in various ways. (Gerson et al., 2006; Luo and Sajda, 2006; Dyrholm et al., 2007; Sajda et al., 2010). The framework showed success in RSVP tasks for triaging image databases of natural scenes (Gerson et al., 2006), aerial images (Parra et al., 2007), and missile detection in satellite images (Sajda et al., 2010). Alpert et al. (2014) presented a a two step linear classification algorithm for RSVP tasks. The Spatially Weighted FLD-PCA (SWFP) algorithm first learns a spatio-temporal weights matrix that amplifies important locations for classification in both spatial and temporal domains. Subsequently, it uses PCA for dimensionality reduction of the temporal domain, and the final features are classified with a FLD classifier. The algorithm was tested on a RSVP task, where the subject was required to detect images of a predefined target category among five categories (eggs, watches, cars, planes, faces) at a display rate of 10 Hz. Despite the difficulty of the task, the algorithm achieved classification performance that is suitable for real life applications.

Many of the BCI algorithms, including those described above, mainly use linear algorithms. Linear methods are simple and fast to train due to their linear constraint which makes them more robust to overfitting. This usually makes linear methods a good choice when dealing with noisy data, such as single trial EEG. On the other hand, the linearity limits the features these algorithms can learn and thus their classification performance is also limited, when the data is not linearly separable. In contrast, non-linear methods can model a wide variety of functions and thus can extract more expressive features, but require careful training to avoid overfitting.

Neural networks are a nonlinear architecture for feature extraction and classification which can learn very complex patterns. Deep, multi-layered, neural networks have achieved break-through results in various tasks such as image classification (Krizhevsky et al., 2012), speech processing (Hinton G. et al., 2012), and action recognition (Ji et al., 2013). These networks have shown to be able to handle large variability in the data, which make them appealing for use with EEG. Specifically, convolutional neural networks (CNNs or ConvNets) (LeCun et al., 2010) appear to be a good fit for EEG data (Cecotti and Gräser, 2011; Manor and Geva, 2015).

Our previous work (Manor and Geva, 2015) introduces a CNN to classify single trial EEG in a RSVP task. The network uses a spatio-temporal regularizer for EEG that reduces overfitting and increases performance. The network was compared to Alpert et al. (2014) using the same data set, showing improved performance.

One of the advantages of neural networks is that all building blocks can be chained together and trained simultaneously on the same objective function. One example of this is building multimodal networks, where multiple modalities of the same information are used to learn better feature representations. In our case of the RSVP task, we have two representations of the object — an image, i.e., pixels, and an EEG brain response corresponding to the image. Therefore, using these two modalities should provide more information to our model and improve performance of the classification task.

Using computer vision algorithms along with EEG learning was suggested previously. Sajda et al. (2010) suggests a two-step system, using computer vision and EEG separately to build a better classifier. The CV system is built from a low-level feature extractors based on a dictionary, and a mid/high-level feature extractors built on specific-domain grammars. The results of this work show a significant improvement when using the two data modalities. Kapoor et al. (2008) uses EEG and computer vision for object categorization, using different types of chosen feature extractors for each modality, and later combining the features linearly for a final decision. Pohlmeyer et al. (2011) suggests a multimodal system for searching images in large datasets. The system involves an iterative process where EEG is first used to flag interesting images and the CV step re-ranks the images.

Another multi-modal BCI system was presented in Putze et al. (2013). Here, the authors present a hybrid EEG and eye tracking system for localizing events in time and space. The user was presented with objects on the screen which were randomly highlighted, while the system detected when and where is the highlighted object. The EEG modality was used to localize the events in time, by using linear EEG features over time windows before and after each highlighting event, which were classified by a Support Vector Machine (SVM) classifier. The spatial location was predicted by the eye tracking system, using the first fixation after a saccade as the target location. Overall, the system showed robust performance for multiple subjects and demonstrated the usefulness of using the two modalities.

We suggest using neural networks for combining computer vision and EEG data. Neural networks allow a natural combination of different features without the need of specific domain knowledge. The modules of the neural network are generic and thus don't require any manual feature engineering. This way, we avoid the need to define specific feature extractors by learning the features from the training data.

Here we present two variants of a multimodal EEG-Image neural network. The first neural network model is designed for RSVP applications where we have a known category of targets in our image data set. In this case, we can train the entire network on EEG and image data simultaneously, in a fully supervised manner. Because we know what the targets look like, we can obtain training samples of target images and train the network to detect these specific targets.

The other variant of the multimodal network is for the case when we don't know a specific category for the target images or that the targets are varied and might change during the RSVP sessions. In this case, it wouldn't be optimal for the network to learn what targets look like, since the network will be required to generalize to unknown target images. Given that the P300 EEG response is agnostic to the type of target that caused the stimuli (Polich, 2007), we need to generalize the image part of the network. For this, we train the image modules of the network as an unsupervised autoencoder (Masci et al., 2011) only on non-target images. Subsequently, we use the features of the autoencoder as the image inputs to the multimodal neural network. Since we don't train on target images at all, the network will be robust to changes in the target images, as long as the non-target images remain similar. By definition, this kind of architecture will have worse performance than the supervised network, but real applications can require this kind of robustness.

We evaluate the suggested models in a RSVP experiment with aerial satellite imagery and compare its performance with each data modality separately. The trained model is then visualized in order to understand what the network has learned.

## 2. Materials and methods

### 2.1. Subjects

Two subjects (one male, one female; mean age 26) participated in a RSVP experiment, both were students of the Hebrew University of Jerusalem, with previous training in aerial imagery analysis. All subjects had normal or corrected to normal vision, with no known neurological problems, were free of psychoactive medications at the time of the experiment, and were paid for their participation. The experiment was approved by the local ethics committee at the Hebrew university of Jerusalem, with written informed consent from all subjects in accordance with the Declaration of Helsinki.

### 2.2. Stimuli

**Figure 2:**
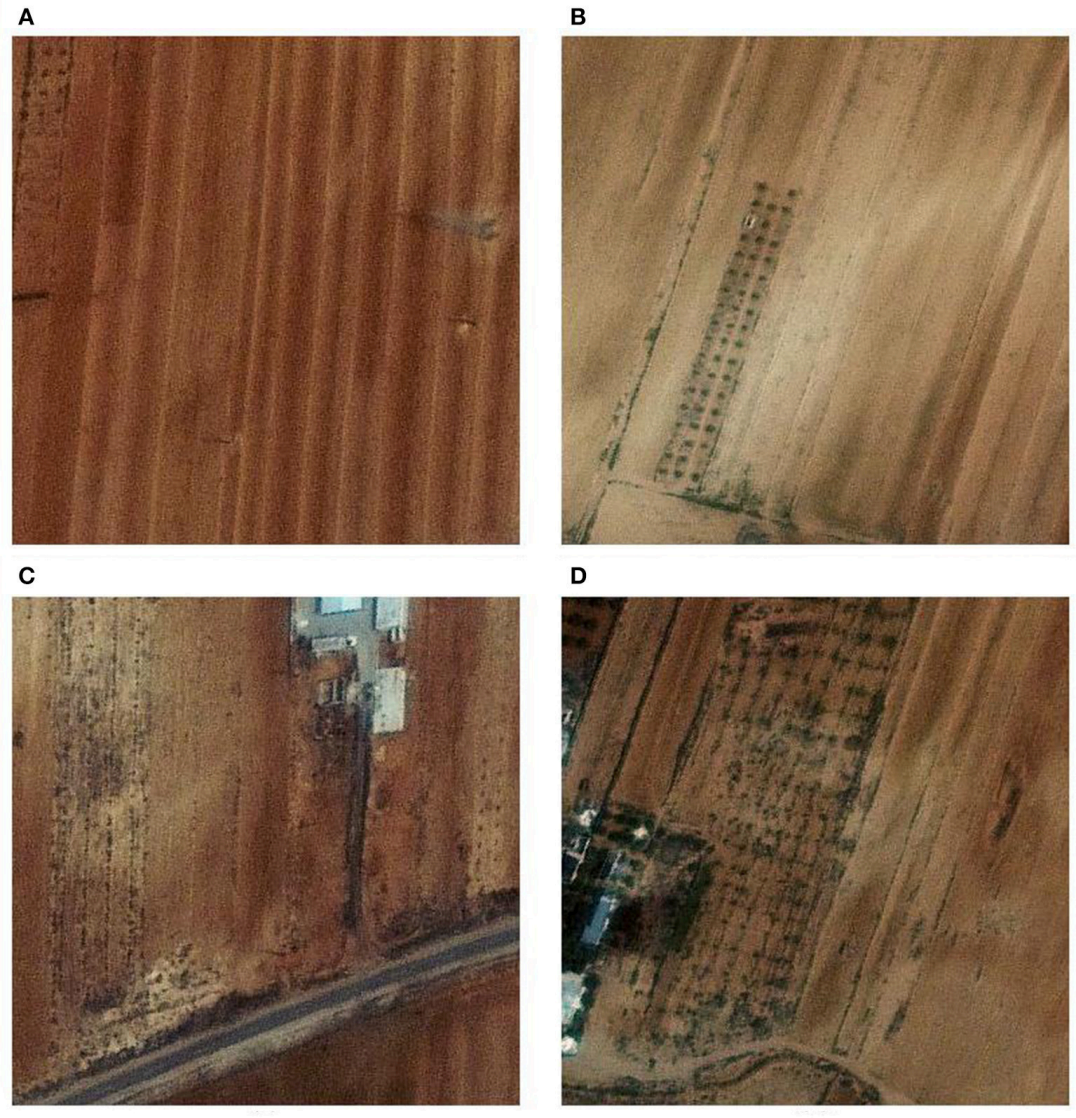
**(A,B)** — Sample non-target stimuli. **(C,D)** — Sample target stimuli.

The stimuli images were made of a color satellite images at a resolution of 0.5 m per pixel, covering a total ground of 100 squared kilometers. The satellite image was divided into images of 400 × 400 pixels with 50% overlap between adjacent images. Overall there were 9411 unique images. The images were displayed at the center of a CRT monitor (Viewsonic model g57f, refresh rate 100 Hz, resolution 1024 x 768) on a gray background. The images were preprocessed to have the same mean luminance and contrast. About 10% of the samples were targets and the rest were non-targets. This ensures that the targets are a rare stimulus among all of the stimuli and a P300 response will be generated when the subject is presented with a target image. Figure 2 presents samples of the stimuli. The target images contain various kinds of structures like buildings or roads. The non-target images do not contain structures but can contain various patterns in the ground because of plants or other natural items. We evaluated the separation of the two classes of images by extracting features from the images using PCA and classifying them using a FLD classifier. The overall correct classification rate was 65.7% with 57.94% hit rate and 33.67% false alarm rate. This indicates that classifying these images require a more capable model, such as the neural network presented ahead.

### 2.3. Experimental procedure

Subjects were seated in a dimly lit, sound attenuated chamber, supported by a chin, and forehead rest. Subjects were instructed to search for buildings in the aerial images. The first two sessions of each subject displayed images at 5 Hz, while the other four sessions displayed images at 10 Hz. Eye position was monitored using an Eyelink 2k/1000 eye tracker (SR research, Kanata, ON, Canada) at 1000 Hz resolution. Presentation was briefly paused every 120–180 image trials for about thirty seconds to avoid cognitive overload. Images were randomly displayed between one to four times in each session. Overall, each session showed about 9000 images.

### 2.4. EEG acquisition and preprocessing

EEG was recorded by an Active 2 system (BioSemi, the Netherlands) using 64 sintered Ag/AgCl electrodes, at a sampling rate of 256 Hz with an online low-pass filter of 51 Hz to prevent aliasing of high frequencies. Additional electrodes were placed as follows: two on the mastoid processes, two horizontal EOG channels positioned at the outer canthi of the left and right eyes (HEOGL and HEOGR, respectively), two vertical EOG channels, one below (infraorbital, VEOGI) and one above (supraorbital, VEOGS) the right eye, and a channel on the tip of the nose. Electrodes were referenced to the average of the entire electrode set, excluding the EOG channels. Offline processing included a band-pass filter of 0.3–20 Hz, and computing bipolar vertical EOG (VEOG) and horizontal EOG channel (HEOG) channels as the difference between VEOGS and VEOGI for VEOG, and the difference between HEOGL and HEOGR for HEOG. The recorded data was segmented to 900 ms segments starting at each image onset. Therefore, each single trial recording yielded a data matrix of 900 ms over 64 channels. The data matrices were downsampled to 64 Hz to reduce computational time, and each dimension of the matrix was normalized to zero mean and variance. The resulting matrix consisted of 64 rows of EEG channels and 64 columns of time samples. We removed the DC baseline from each channel separately. Large artifacts, e.g., blinks, were removed by rejecting trials in which the VEOG bipolar channel exceeded ±100 μ*V*. The images of the stimuli were downsampled to 50 × 50 pixels before using them as input to the network.

### 2.5. Neural network architectures

Our neural network model receives as input an image and a single-trial EEG data matrix, corresponding to the brain response of the image. We wish to train the network to classify these inputs into one decision, targets or non-targets. This brings the question of how to fuse together the inputs to get one classification result. One option is to train directly on the concatenated image and EEG data, i.e., input-level fusion. In this case, the first layer of the network has to learn the correlations between the inputs and select the appropriate features. However, the relation between the different modalities can be non-linear, and so it can be hard for the network to learn these directly (Ngiam et al., 2011). Decision-level fusion (Atrey et al., 2010) suggests making a decision for each input separately, and then combine the decisions. The advantage here is that each decision has the same representation which makes it easy to combine and add more decisions later if needed. On the other hand, decision-level fusion doesn't use correlations of the features of the different modalities and therefore might miss important information. The third option, which we use in this work, is feature-level fusion. In this case, the network learns features separately for each type of data, and later joins these features for the final layers. This allows the network more freedom in transforming each modality of the data before trying to learn the interactions between them. This also allows the network to use features from both modalities to reach a decision when each one of the modalities would not have enough support. For example, very noise inputs might not be able to be detected on their own, but each of the inputs can contribute to an overall successful detection.

#### 2.5.1. Supervised model

The supervised model addresses the case where the target category is known in advance, e.g., detecting people in natural scenes. This allows us to use target images for training since future target images will have the same type of targets.

It is possible to train separate networks for images and EEG, and then train another network to combine the results into a final decision. However, training on both inputs simultaneously will jointly optimize all features so that they will work better together for classification. In general, end-to-end training was shown to be a better choice over separate training steps in a machine learning pipeline (Hannun et al., 2014; Levine et al., 2015).

As noted earlier, we join the features of the images and EEG in a deep layer in the network. Our network starts with separate layers for each input, continues with a layer that concatenates features from each input and ends with a final classification layer.

**Figure 3:**
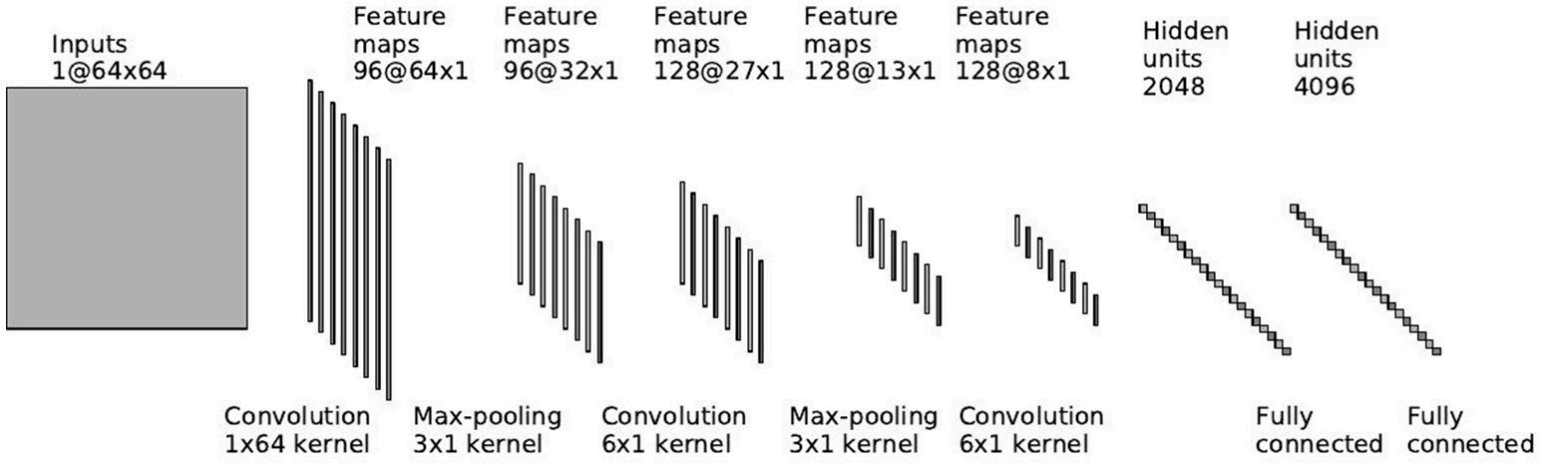
**EEG network architecture**.

The EEG layers of the network follow our network in Manor and Geva (2015), without the output layer. The input to the EEG network is a single trial matrix of 64 electrodes by 64 time samples. The network contains three convolutional layers, two pooling layers and two fully connected layers. The first convolutional layer performs a spatial convolution by using filters of size 64 × 1, learning features which represent a spatial distribution across the scalp. Since this layer is convolutional, the weights of the filters are shared across time and it is insensitive to temporal latencies. The second layer is a max-pooling layer (LeCun et al., 2010) to reduce dimensionality. The pooling filters are in size of three samples and with stride of two samples. Therefore, we reduce the dimensionality, but we still have overlap to avoid losing information (Le Cun et al., 1990). The max operation provides a small invariability in the temporal domain, as long as the samples stay within the same pooling filter. The following layers are a temporal convolutional layer, with filter size of about 100 ms, following another max-pooling layer and another temporal convolutional layer of the same size. The last two layers of the network are dense, fully-connected layers, of sizes 2048 and 4096. Figure 3 shows an overview of the network. Please see Manor and Geva (2015) for more details.

**Figure 4:**
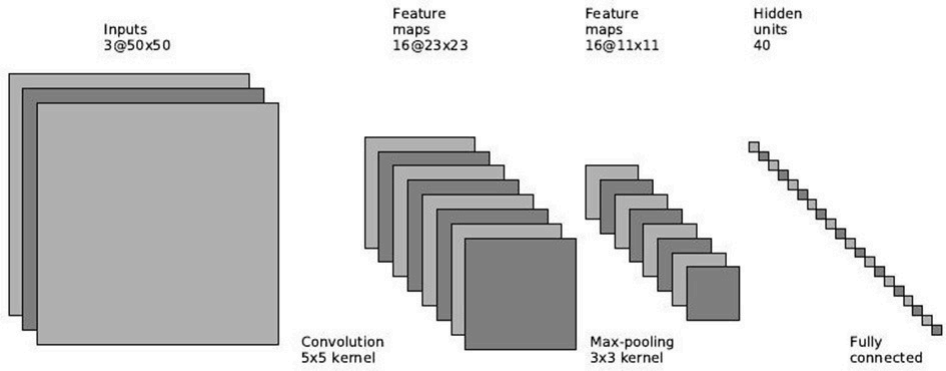
**Supervised image network architecture**.

The image network follows the common architecture for object recognition (Krizhevsky et al., 2012; LeCun et al., 2010). We use a convolutional layer with 5 × 5 filters with 2 × 2 pixels stride, followed by a pooling layer with 3 × 3 filters and the same stride. The final features layer is a fully connected layer that outputs 40 features representing the image. Figure 4 depicts the supervised image network architecture.

Both networks use the ReLU non-linearity (Nair and Hinton, 2010), f(x) = max(0, x), after each convolutional and fully-connected layer. Dropout (Hinton G.E. et al., 2012) is used on each fully-connected layer to decrease overfitting.

**Figure 5:**
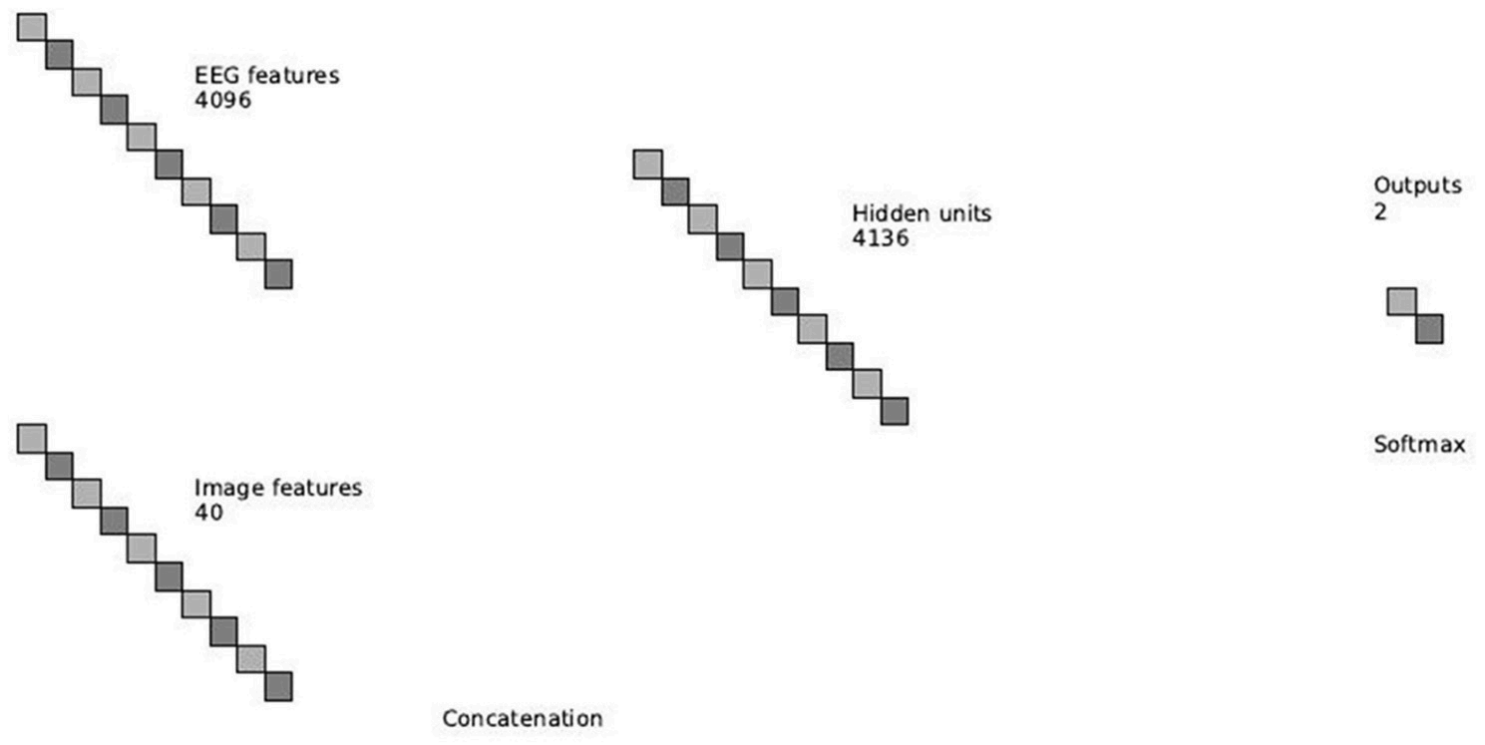
**Overview of the multimodal neural network**.

The features from the above networks are concatenated into one feature vector, which is then fed into a final softmax classification layer. Figure 5 depicts an overview of the combination of the EEG and image features.

The entire network is trained by minimizing the multinomial logistic regression loss function:

(1) $$\begin{array}{l} L=-\overset{N_{samples}}{\underset{i}{\sum }}\log\left[\left(1-y^{\left(i\right)}\right)h_{0}\left(x^{\left(i\right)}\right)+y^{\left(i\right)}h_{1}\left(x^{\left(i\right)}\right)\right] \end{array}$$

where *N*_*samples*_ is the number of training samples, *x*^(*i*)^ is the *i* training sample, *y*^(*i*)^ is the true label of sample *i* and *h*_*k*_ is the neural network output unit *k*.

#### 2.5.2. Semi-supervised model

Some RSVP applications has to deal with a wide variety of targets or even unknown targets, e.g., detecting various items in x-ray images (Trumbo et al., 2015). In this case, we can't train on the target images since this will cause the network to detect only targets that are present in the training set. To overcome this limitation, we suggest using an unsupervised autoencoder model for the images, and later join with the EEG network in the same method described above. An autoencoder (Masci et al., 2011) is an unsupervised model that is trained to reconstruct the input from extracted features. This is similar to PCA, but an autoencoder can be composed of multiple nonlinear functions, unlike PCA which is linear.

**Figure 6:**
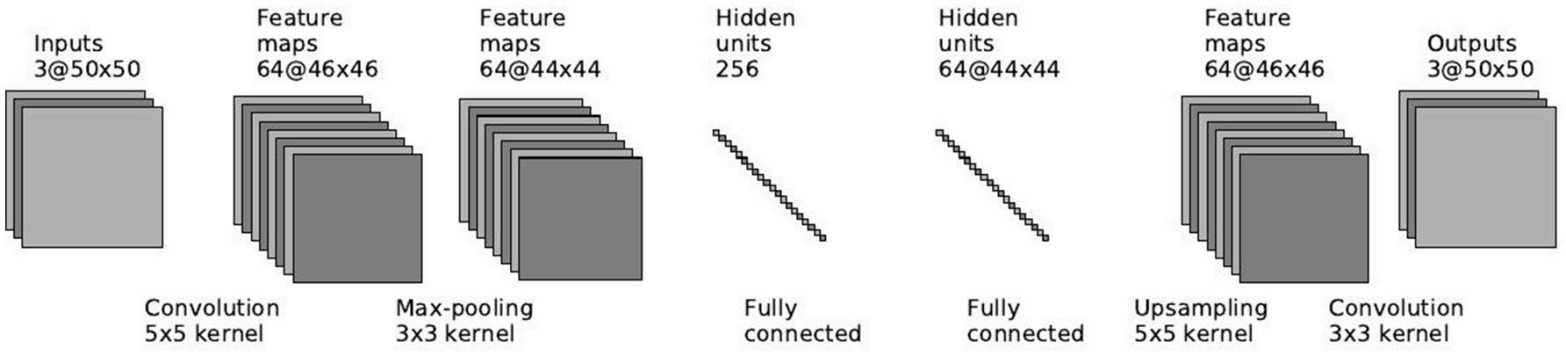
**Image autoencoder architecture**.

Our autoencoder model is based on the supervised image network presented in the previous section. The autoencoder starts with a convolutional layer with 5 × 5 filters, followed by a pooling layer with 3 × 3 filters. The final encoding layer is a fully connected layer with 256 units. The decoding part of the network is built the same way in reverse order: a fully connected layer, followed by a reverse max pooling layer (up-sampling), and a convolutional layer. ReLU non-linearities are used after each convolutional and fully-connected layer. Figure 6 depicts the architecture of the autoencoder.

The autoencoder is trained by minimizing the mean squared error loss function:

(2) $$\begin{array}{l} L=\overset{N_{samples}}{\underset{i}{\sum }}{\left(x^{\left(i\right)}-h^{\left(i\right)}\right)}^{2} \end{array}$$

Note that only the input *x* and the output *h* are used to train the image autoencoder, without using the labels.

When training the multimodal network, we first train the image autoencoder only on non-target images. This simulates a real use case where we do not know how the targets will look in experiments. The autoencoder learns features that models that non-target images, and even though it doesn't use labels, it should output different features for non-target and target images, since they actually look different in the pixel level. The features that are output from fully connected layer are used as inputs for multimodal neural network, instead of the original images.

### 2.6. Learning parameters

Both networks use stochastic gradient descent (SGD) for minimizing the loss function. We use a decaying learning rate computed by *lr* = 0.0001*0.99^*n*^ where *n* is the epoch number. We also used a constant momentum of 0.9 (Polyak, 1964).

The momentum update *V* is computed with

(3) $$\begin{array}{l} V=\gamma V+\eta Q \end{array}$$

where η is the learning rate, γ is the momentum coefficient and *Q* is the gradient of the loss function with respect to the network parameters. The parameters *W* are then updated with

(4) $$\begin{array}{l} W=W-V \end{array}$$

The gradient *Q* is computed with the classical back-propagation algorithm.

It should be noted that computing the gradient using a single sample, instead of using a mini-batch, accelerated the convergence time of the network. The values of the learning parameters were chosen empirically by cross-validation. The cross validation was performed on a randomly selected data set, which was then randomly split into training and validation sets. Each parameter value was tested on ten runs of the random training and validation selection process. The values of the parameters were chosen manually. The whole process was performed before the actual classification and the parameters values were held constant for all subsequent runs.

### 2.7. Class imbalance

The nature of our experiment causes the data classes to be highly imbalanced, as only 10% of the samples are targets. Gradient descent methods do not perform well on unbalanced datasets because the gradients will follow the majority class. To overcome this bias, we bootstrapped the targets class to match the size of the non-targets, only in the training set (Manor and Geva, 2015). Although, this caused some overfitting on the target class, it provided an overall more balanced classification performance in our experiments.

## 3. Results

### 3.1. Classification performance

We tested our networks using a random cross-validation procedure, separately on each session per subject. The dataset was randomly split into a 80% training set and a 20% test set repeatedly. The presented results are an average of the test performance of ten runs on random train/test splits. All experiments were implemented with Lasagne (Dieleman et al., 2015) on a NVIDIA GTX 650 GPU.

The supervised network was trained from scratch in each cross-validation permutation, on the randomly chosen training set. For the semi-supervised network, we first perform the random selection of training and test sets. Then, we trained the auto-encoder only on non-target samples from this training set, and used the entire training set to train the entire network, with the autoencoder features used instead of the images.

Classification results are summarized in Table 1 for the supervised network and Table 2 for the semi-supervised network. The performance is presented in terms of correctly classified single trials, hit rate (true positive rate) and false alarm rate (false positive rate), where positive is the target class. The correct classification metric is defined as the sum of correct positives and negatives among all samples, and therefore can show a distorted view of performance due to the imbalanced classes in our data set, i.e., if all trials are classified as non-target, we get 90% correct classification. Therefore, we also compute the Area Under the Curve (AUC) and balanced accuracy metrics. The balanced accuracy is defined as $\frac{\text{true positive rate}\;+\;\text{true negative rate}}{2}$ and thus is a more suitable metric for our case. The standard deviations in the table and figures are across the repeated cross validation test sets, except for deviations at the mean row which are across subjects.

**Table 1:** **Supervised network classification results**.

**Subject/Session**	**Correct**	**Hits**	**False alarms**	**AUC**	**Balanced Accuracy**
A-1	90.07 ± 1.1	78.22 ± 4.3	9.17 ± 1.4	0.91 ± 0.02	84.52 ± 4.3
A-2	88.14 ± 2.1	79.86 ± 4.6	11 ± 2.2	0.92 ± 0.03	84.33 ± 4.6
A-3	88.48 ± 2.7	78.48 ± 3.4	10.74 ± 3	0.93 ± 0.01	83.87 ± 3.4
A-4	91.89 ± 2.7	81.26 ± 3.4	7.32 ± 3	0.94 ± 0.02	86.96 ± 3.4
A-5	93.89 ± 2.1	77.7 ± 3	4.99 ± 2.2	0.94 ± 0.02	86.35 ± 3
A-6	90.15 ± 1.6	75.52 ± 3.7	8.6 ± 1.8	0.92 ± 0.02	83.46 ± 3.7
B-1	93.1 ± 1.7	82.32 ± 1.8	6.37 ± 1.4	0.94 ± 0.02	87.97 ± 1.8
B-2	89.96 ± 2.9	77.27 ± 4	8.93 ± 3.2	0.91 ± 0.03	84.17 ± 4
B-3	92.65 ± 1.6	79.56 ± 4.2	6.4 ± 1.8	0.93 ± 0.03	86.57 ± 4.2
B-4	88.09 ± 2.2	76.34 ± 3.4	10.98 ± 2.3	0.9 ± 0.02	82.67 ± 3.4
B-5	90.74 ± 1.3	76.53 ± 5	8.06 ± 1.6	0.92 ± 0.03	84.23 ± 5
B-6	92.86 ± 1.7	77.3 ± 3.2	6.07 ± 1.8	0.92 ± 0.03	85.61 ± 3.2
Mean	90.84 ± 2	78.35 ± 2	8.22 ± 2	0.92 ± 0.01	85.06 ± 1.6

**Table 2:** **Semi-supervised network classification results**.

**Subject/Session**	**Correct**	**Hits**	**False alarms**	**AUC**	**Balanced accuracy**
A-1	89.91 ± 0.6	75.28 ± 3.2	8.95 ± 0.8	0.90 ± 0.03	83.16 ± 1.7
A-2	84.59 ± 1.3	76.01 ± 2.5	15.14 ± 1	0.87 ± 0.02	80.43 ± 1.4
A-3	81.64 ± 1	73.61 ± 2	17.65 ± 1.1	0.87 ± 0.02	77.97 ± 1.2
A-4	86.35 ± 1.2	70.58 ± 3.5	12.6 ± 1.3	0.86 ± 0.03	78.98 ± 1.8
A-5	88.16 ± 1.5	71.58 ± 2.6	10.77 ± 1.5	0.89 ± 0.02	80.40 ± 1.7
A-6	84.62 ± 0.9	75.21 ± 1.8	14.65 ± 0.7	0.89 ± 0.03	80.28 ± 0.9
B-1	90.29 ± 1	73.43 ± 2.2	8.57 ± 1	0.87 ± 0.04	82.43 ± 1.3
B-2	84.7 ± 1.3	73.89 ± 4	14.57 ± 1.2	0.86 ± 0.03	79.66 ± 2.4
B-3	87.92 ± 1.7	74.09 ± 5.1	11.07 ± 1.8	0.90 ± 0.02	81.51 ± 2.9
B-4	81.44 ± 1.5	78.09 ± 3.6	18.24 ± 1.6	0.88 ± 0.03	79.92 ± 2
B-5	84.23 ± 1.2	75.04 ± 2.4	15 ± 1.3	0.90 ± 0.03	80.01 ± 1.5
B-6	90 ± 0.9	75.73 ± 3.2	8.96 ± 0.9	0.91 ± 0.03	83.38 ± 1.6
Mean	86.15 ± 3.1	74.38 ± 2.2	13.01 ± 1.4	0.88 ± 0.01	80.68 ± 1.1

The supervised network achieved 0.9–0.94 AUC across subjects (mean: 0.92; std: 0.01) with balanced accuracy of 83–87% (mean: 85.06%; std: 1.6%). In comparison, the semi-supervised network results are lower. This is expected since the semi-supervised network does not learn the target images and thus has a more difficult task. The network achieved 0.86–0.91 AUC (mean: 0.88; std: 0.01) with balanced accuracy of 77–83% (mean: 80.68%; std: 1.1%).

We examine the differences in performance between the two presentation rates, 5 Hz (sessions 1–2) and 10 Hz (sessions 3–6). Overall we do not see a significant performance changes. In the supervised network, the averaged balanced accuracy didn't change for subject A (84.42 vs. 85.16%) while for subject B there was a minor drop (86 vs. 84.77%). In the semi-supervised network, the averaged balanced accuracy for subject A decreased from 81.79% for the 5 Hz sessions to 79.41% for the 10 Hz sessions, while subject B had no change (81 vs. 81.21%).

**Figure 7:**
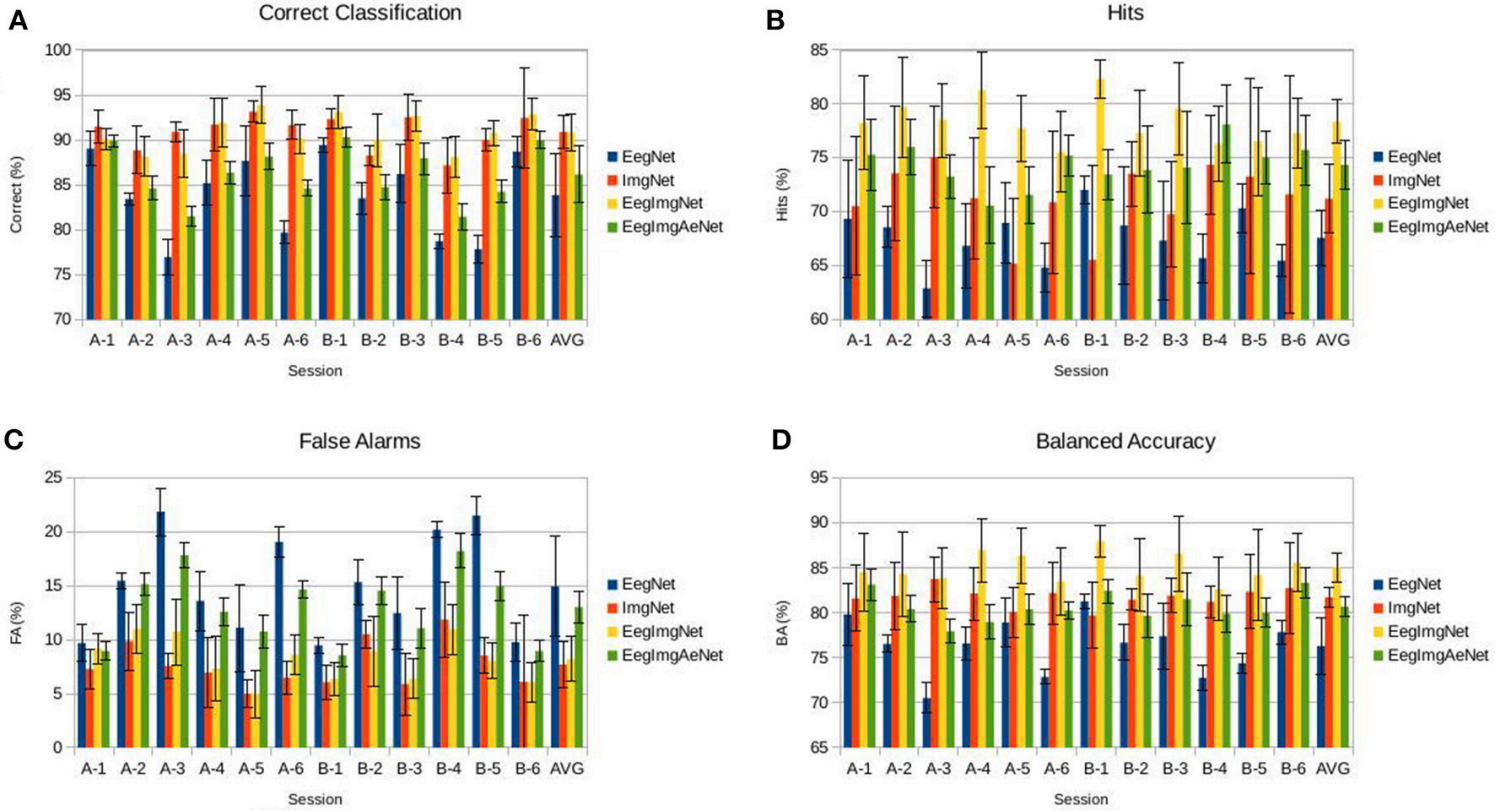
**Classification performance of four neural networks - EegNet (EEG data only), ImgNet (image data only), EegImgNet (supervised EEG and image network), EegImgAeNet (semi-supervised EEG and image network). (A)** Correct classification across subject. **(B)** Hit rate (true positive rate). **(C)** False alarm rate (false positive rate). **(D)** Balanced Accuracy.

We compare performance of the multimodal networks and each of the modalities separately in Figure 7; EegImgNet is the supervised network and EegImgAeNet is the semi-supervised network.

The supervised multimodal network is superior than using each one of them separately. We can see a significant increase in hits, where the multimodal network improves in +7% over the image network and +11% over the EEG network, and in balanced accuracy where the combined network has +4% over the image network and +9% over the EEG network.

The semi-supervised multimodal network is always better than the EEG network, showing on average a +3% improvement in correct classification, +7% in hits, −1% in false alarms, and +4% in balanced accuracy.

Comparing the EEG and image networks, the images are almost always superior, except for one session (B-1). Nevertheless, the fact that the supervised multimodal network shows an improvement over both modalities means that each modality, EEG and images, has its own strength, as indicated by the strong improvements in the hits metrics.

We used non-parametric tests to to assess the significance of the changes in the performance metrics between the all networks simultaneously. Kruskal–Wallis (Kruskal and Wallis, 1952) was used to determine the differences between all networks on the various metrics. All *p*-values were <0.05, as seen in Table 3. We can account for the multiple tests with Bonferroni correction (Dunn, 1961), which adjusts *p* to $p=\frac{\alpha }{m}=\frac{0.05}{5}=0.01$. The results of the Kruskal–Wallis tests are still below the adjusted *p*-value.

**Table 3:** **Kruskal–Wallis test *p*-values**.

**Correct**	**Hit rate**	**False alarm**	**AUC**	**Balanced accuracy**
1.2e-5	5.4e-8	1.1e-5	2.6e-7	4.9e-8

Wilcoxon signed rank test (Wilcoxon, 1945) was used to assess the pair-wise changes between the networks. Table 4 presents the *p*-values for all tests. For multiple testing correction, we use Holm-Bonferroni (Holm, 1979) correction with α = 0.05 and *m* = 30. After the correction, the tests determined a significant change when comparing both multimodal networks with the EEG network on all metrics. Comparing the supervised multimodal network with the image network, the test pointed out a significant change in the hits and balanced accuracy metrics. The semi-supervised multimodal network was significantly different than the image network on the correct, hits, and false alarms metrics.

**Table 4:** **Wilcoxon test *p*-values (correct / hits / false alarms / AUC / balanced accuracy)**.

	**EegNet**	**ImgNet**	**EegImgNet**	**EegImgAeNet**
EegNet	–	4e-4 / 0.05 / 4e-4 / 0.001 / 0.001	4e-4 / 4e-4 / 4e-4 / 4e-4 / 4e-4	4e-4 / 4e-4 / 4e-4 / 9e-4 / 4e-4
ImgNet	–	–	0.79 / 4e-4 / 0.26 / 0.01 / 4e-4	4e-4 / 0.006 / 4e-4 / 0.12 / 0.15
EegImgNet	–	–	–	4e-4 / 0.003 / 9e-4 / 4e-4 / 4e-4

To verify our choice of a feature-level fusion model, we compare the results of the supervised network to another supervised network, where the fusion of the modalities occurs in the decision-level. Namely, each modality has its own classifier that outputs a decision, and another classifier combines both decisions into a final one. Table 5 depicts the performance of the decision-level fusion network. We can see that on average every metric is lower with decision-level fusion, with the biggest decrease in hits and balanced accuracy, which dropped in 4%.

**Table 5:** **Decision-level fusion supervised network classification results**.

**Subject/Session**	**Correct**	**Hits**	**False alarms**	**AUC**	**Balanced accuracy**
A-1	87.62 ± 2.11	76.72 ± 2.01	11.78 ± 2.26	0.9 ± 0.02	82.47 ± 1.65
A-2	86.59 ± 1.14	74.2 ± 2.55	12.24 ± 0.97	0.89 ± 0.03	80.98 ± 1.24
A-3	85.45 ± 2.16	71.43 ± 2.3	13.41 ± 2.53	0.88 ± 0.03	79.01 ± 1.2
A-4	85.07 ± 3.38	77.51 ± 3.86	14.51 ± 3.61	0.91 ± 0.03	81.5 ± 2.35
A-5	91.38 ± 1.56	74.43 ± 3.25	7.49 ± 1.69	0.92 ± 0.03	83.53 ± 1.75
A-6	89.08 ± 1.55	73.42 ± 2.34	9.56 ± 1.8	0.94 ± 0.02	81.77 ± 0.72
B-1	88.87 ± 3.65	77.68 ± 3.96	10.38 ± 3.96	0.92 ± 0.03	83.55 ± 2.89
B-2	87.74 ± 1.51	75.77 ± 4.52	11.31 ± 1.83	0.9 ± 0.05	82.2 ± 2.2
B-3	88.77 ± 2.82	72.86 ± 3.68	10.13 ± 3.11	0.9 ± 0.04	81.47 ± 2.36
B-4	86.83 ± 1.6	74.53 ± 5.93	12.1 ± 1.9	0.92 ± 0.02	81.62 ± 2.86
B-5	87.82 ± 1.99	74.47 ± 2.4	11.09 ± 2.09	0.91 ± 0.03	81.89 ± 1.78
B-6	91.96 ± 1.19	73.84 ± 3.12	6.85 ± 1.2	0.9 ± 0.03	83.48 ± 1.83
Mean	88.1 ± 2.09	74.74 ± 1.88	10.9 ± 2.22	0.91 ± 0.02	81.96 ± 1.28

### 3.2. Features analysis

We inspect the weights that were learned by the supervised multimodal network and the features that were extracted. For the EEG part, although we didn't restrict the network to learn specific P300-related features, we still expect to find some resemblance to the P300 ERP distribution. For the images, we wish to see which structures were identified as targets.

**Figure 8:**
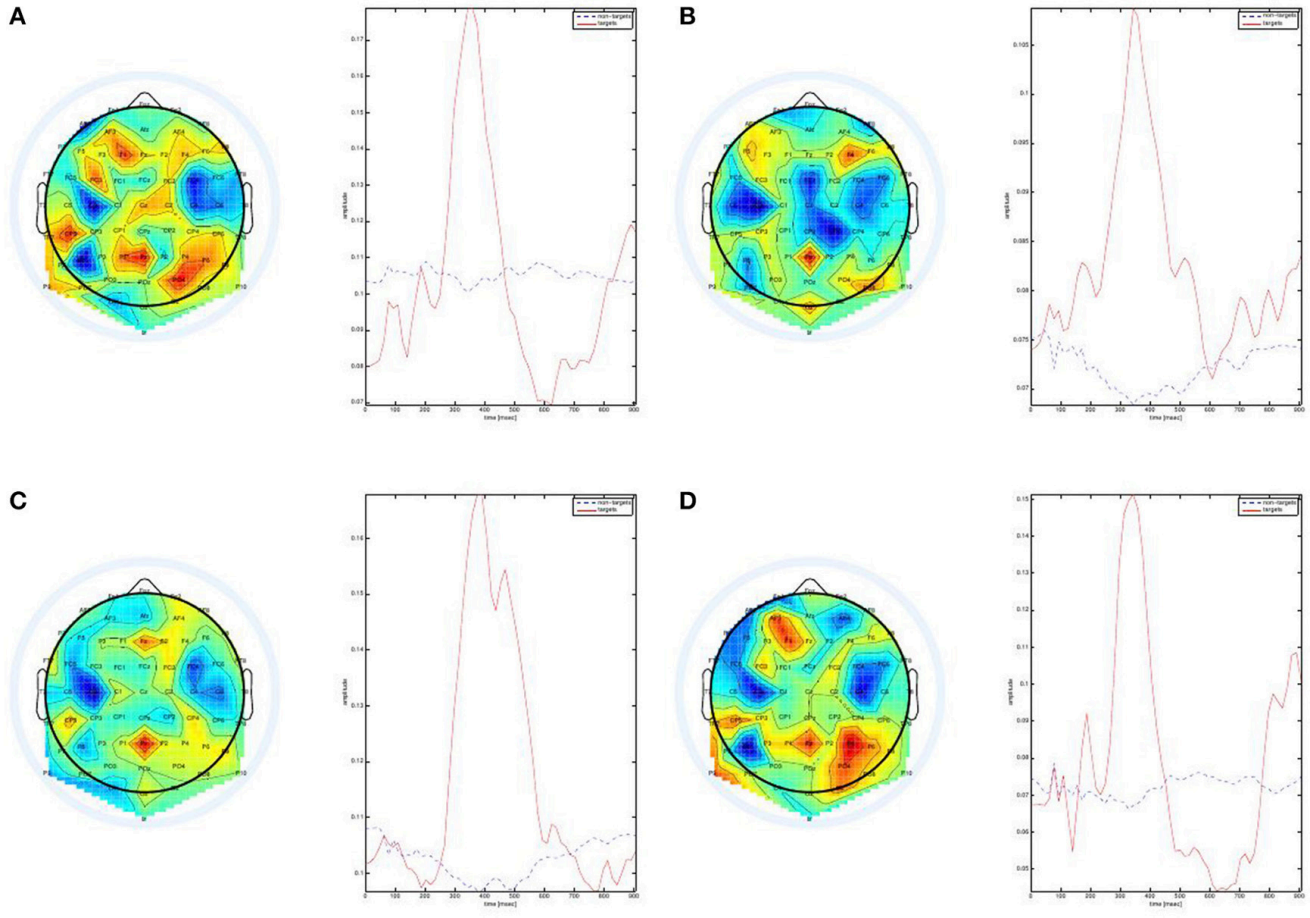
**Weights of the first convolutional layer in the EEG network**. All samples show high activity around Pz which is typical to P300 ERPs. All scalp plots were generated using EEGLAB (Delorme and Makeig, 2004).

We start by plotting the weights and features of the first EEG convolutional layer. The weights of this layer captures only the spatial values of the electrodes while the convolution is performed across time. Therefore, the features emitted by this layer can be thought of as the amplitude of the weights in time. Figure 8 shows a sample of four weights vectors, plotted on the scalp, and their corresponding temporal activations.

The scalp plots show high positive activity in frontal and parietal electrodes. This is inline with the known P300 distribution on the scalp (Polich, 2007). In addition, we see that the maps are mostly active around 300–500 ms, which is also as expected. Figure S4 in the Supplementary Material shows additional maps from both subjects, where we can see additional spatial patterns that were learned by the network.

The presented weights show us a partial picture of what the network has learned since we do not see the affect of the deeper layers of the network. Therefore, we use a gradient-based method (Simonyan et al., 2013) to generate saliency maps of a given input sample. The maps are generated from back-propagating gradients via the entire network, yielding a complete image of what the network has detected in the inputs. We can only use it for the supervised multimodal network since it is fully trained with one loss function, unlike the semi-supervised network.

**Figure 9:**
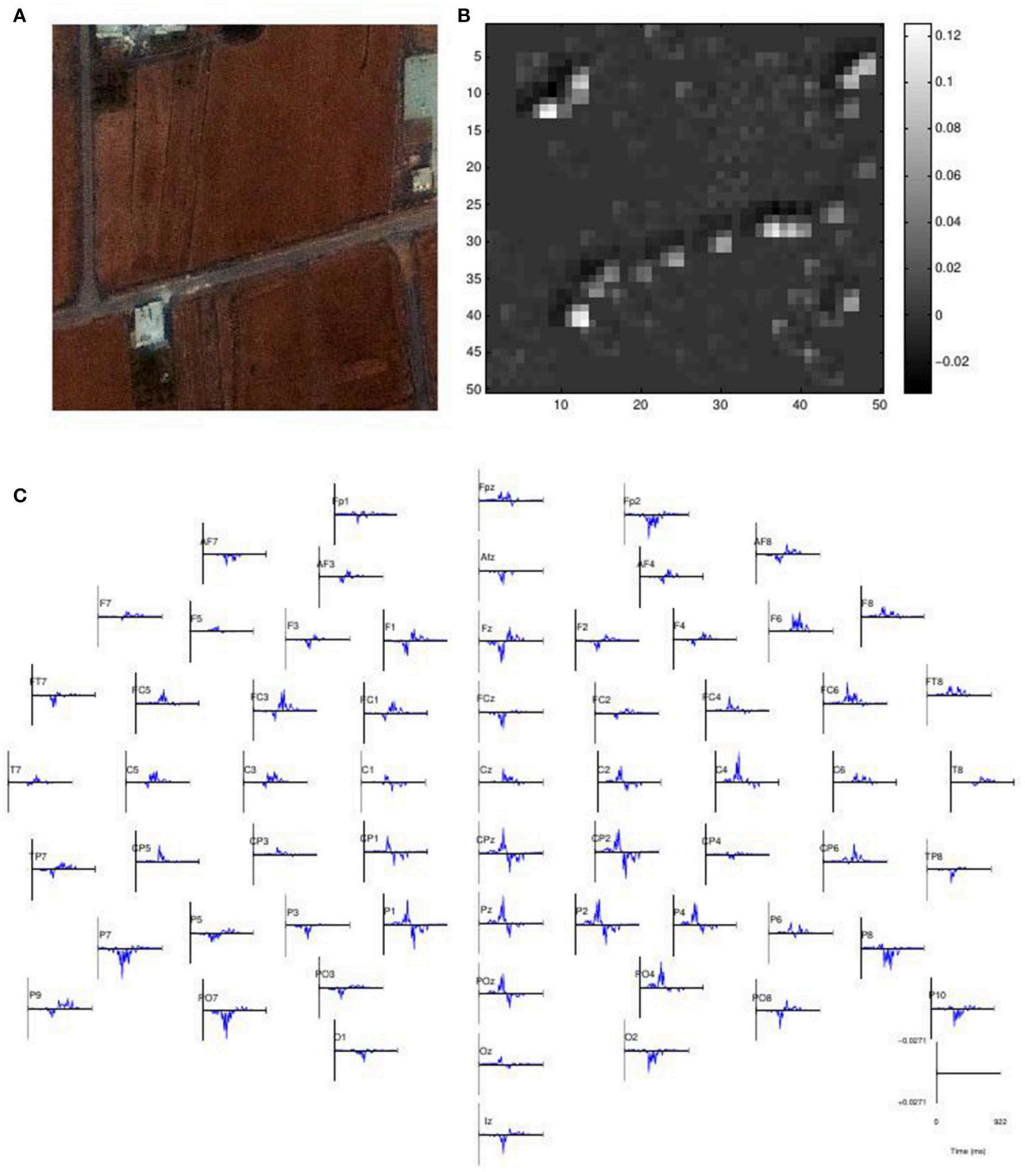
**Visualization for a Sample Input. (A)** The input image. **(B)** Image saliency map. **(C)** EEG saliency map scalp plot.

Figure 9 shows the visualization of a sample input. The input image is a target image with multiple buildings and roads. The image saliency map shows that the network detected three buildings in the image and the horizontal road. The EEG saliency map is shown below, where we see a temporal plot for each channel plotted on the scalp. We can see here that the network detected positive activity in parietal and central channels such as Pz and Cz. Frontal channels such as Fz also have a similar positive activity but later in time. We show the visualizations of additional samples in the Supplemental Data Section, in Figures S1–S3.

## 4. Discussion

BCI applications make heavy use of machine learning to learn and decode brain activity robustly. BCI applications for healthy users are designed to assist users with high throughput tasks by taking advantage of computer algorithms and the brain processing power. Specifically, RSVP applications assist users in scanning large image datasets for a set of target images. These type of applications have visually or auditory stimuli and rely on the user to process them while the BCI application is used to quickly identify the corresponding signals in the user's brain. However, with recent advancements in computer vision, we are inclined to use the computer for processing the stimuli in addition to handling the EEG signal. Here, we present two multimodal neural networks that uses the recorded EEG and the image stimulus simultaneously to improve performance in this task. The first network is a fully supervised model, that is trained simultaneously on EEG and image inputs. The network learns to detect the target-related response in the EEG while also learning how the targets look like in the images. We see that in comparison to the separate EEG or image networks, the supervised multimodal has a much better classification performance across subjects and metrics. When comparing the separate image and EEG networks, we see that the image network has on average better performance than the EEG network. This might indicate that the images in question were “too easy” for the network, and our next experiment should use harder targets in order to emphasize the strength of the EEG features. We also asses our choice of feature-level fusion of the two modalities with decision-level fusion for the supervised network. The results demonstrate that feature-level fusion is beneficial.

The supervised multimodal network learns to detect the P300 ERP in the data, which is in general agnostic to the actual type of the target image since it is elicited when the brain detects a rare stimulus. The images, however, contain specific targets, e.g., structures and buildings. Therefore, if the target images change, we need to retrain our network on a new training set. Another case could be that the targets are unknown at the training stage or might change once we deploy our BCI application to production. For this use case, we introduce a semi-supervised multimodal neural network. Here, we train the network in two steps. First, we train an unsupervised autoencoder on non-target images alone. The model learns how to reconstruct the images from the features, and therefore produces features that represent critical information about the image. After this, we train the multimodal neural network by giving it EEG data as one input, and the image features extracted by the autoencoder as a second input. In this training step, we use both target and non-target images, but since the autoencoder is already trained here, then the entire network does not learn any features from the target images.

The performance of the semi-supervised model is lower than the supervised model and image network. This is expected since the network that sees the target images have more information for classification. Still, the semi-supervised model surpasses the EEG network on every metric across all sessions. This points out that even if the network didn't see the targets, it can still produce meaningful features that can differentiate non-target images and other images that it wasn't trained on. This is an interesting result that can be useful in many RSVP applications.

Multimodal algorithms for EEG have been proposed before (Sajda et al., 2010), using classical computer vision algorithms together with advanced ML algorithms for EEG processing. The advantage of this approach is that it requires little training data since the features are manually engineered by the choice of algorithms. However, this mechanism might fail when the images get more complex and the targets are harder to detect. Here we use a deep neural network to learn features from a training set, a method proven to succeed in difficult object recognition competitions (Krizhevsky et al., 2012). Although it requires training data to learn from, it reduces the amount of design complexity since we do not need to select image-specific algorithms for feature extraction. Therefore, we can easily adapt our model to new, more difficult, images. In addition, since we use similar architectures for the EEG and image networks, we can train them simultaneously and have a performance gain by allowing the two sub-networks to influence each other in the training process.

Recent advancements in machine learning have brought automatic recognition capabilities to real world performance in various domains such as images, speech, and text. BCI applications should use these new algorithms in order to combine information from the brain and from the stimuli presented.

## Author contributions

RM—performed all of the research, implementation and experimentation, guided LM in the visualization section, and wrote the entire paper. LM—performed the visualization and produced the visualizations images. AG—Advisor, supervisor of research, head of lab.

### Conflict of interest statement

The authors declare that the research was conducted in the absence of any commercial or financial relationships that could be construed as a potential conflict of interest.
