# Supplementary material for: Multimodal Neural Network for Rapid Serial Visual Presentation Brain Computer Interface
Source: Front Comput Neurosci. 2016 Dec 20;10:130. doi: 10.3389/fncom.2016.00130 (PMC5168930; doi:10.3389/fncom.2016.00130)
Supplement: Supplementary file 1 [file Image1.pdf]

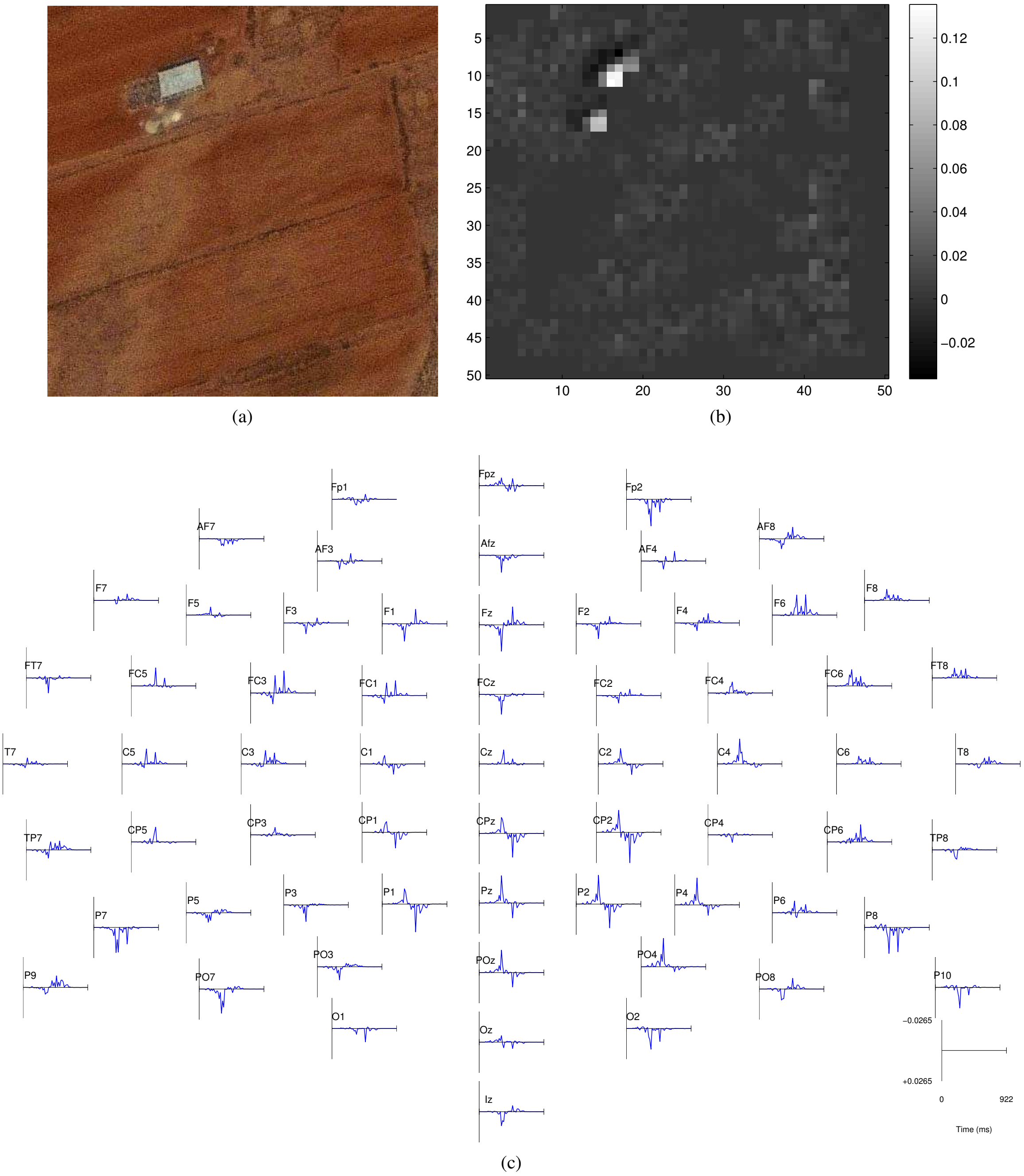

Figure S1. Visualization for a sample input. (a) the input image. (b) image saliency map. (c) EEG saliency map scalp plot.
