# Supplementary material for: Multimodal Neural Network for Rapid Serial Visual Presentation Brain Computer Interface
Source: Front Comput Neurosci. 2016 Dec 20;10:130. doi: 10.3389/fncom.2016.00130 (PMC5168930; doi:10.3389/fncom.2016.00130)
Supplement: Supplementary file 4 [file Image4.pdf]

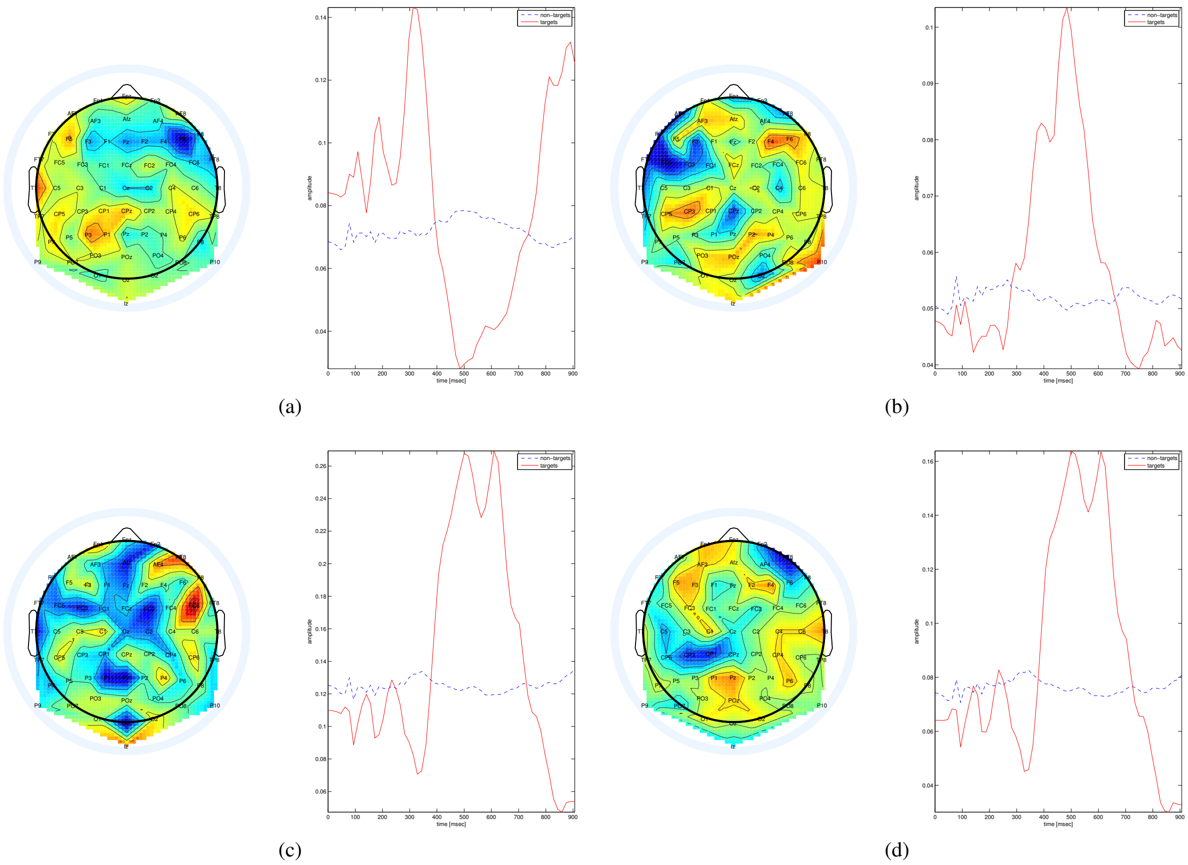


**Figure S4.** Weights of the first convolutional layer in the EEG network from several subjects/sessions, where we can see the variability across the data.
